# 肺腺癌吉非替尼获得性耐药相关microRNAs的筛选鉴定

**DOI:** 10.3779/j.issn.1009-3419.2011.06.18

**Published:** 2011-06-20

**Authors:** 学博 秦, 彬 刘, 洋 李, 嘉琮 尤, 清华 周

**Affiliations:** 300052 天津，天津医科大学总医院天津市肺癌研究所，天津市肺癌转移与肿瘤微环境重点实验 Tianjin Key Labotatory of Lung Cancer Metastasis and Tumor Microenvironment, Tianjin Lung Cancer Institute, Tianjin Medical University General Hospital, Tianjin 300052, China

**Keywords:** 肺肿瘤, microRNA, 吉非替尼, 获得性耐药, Lung neoplasms, microRNAs, Geftinib, Aquired-resistance

## Abstract

**背景与目的:**

肺腺癌吉非替尼获得性耐药严重影响了肺癌的治疗效果，microRNA在肺腺癌吉非替尼获得性耐药中的作用及其机制尚不清楚。本研究筛选与肺腺癌获得性吉非替尼耐药相关的microRNAs。

**方法:**

以吉非替尼敏感肺癌细胞PC9与吉非替尼耐药肺癌细胞PC9/AB11为细胞模型，观察二者的形态学差异，流式细胞仪检测二者的细胞周期，计算它们的倍增时间，MTT法检测吉非替尼对两种细胞的IC_50_，应用microRNA芯片检测和筛选与吉非替尼耐药相关的microRNAs，并进行real-time PCR验证。

**结果:**

PC9细胞与PC9/AB11细胞形态差异明显，在细胞周期、倍增时间和吉非替尼对其的IC_50_上均具有统计学差异。microRNA芯片结果显示，与PC9相比，耐药肺癌细胞株PC9/AB11中有4个microRNAs表达水平明显上调，有9个microRNAs表达水平明显下调。经real-time PCR验证，microRNA-138在PC9/AB11中表达明显下调，与芯片结果一致。

**结论:**

PC9和PC9/AB11细胞株microRNA表达谱存在明显差异，初步筛选到了13个与肺腺癌吉非替尼耐药密切相关的microRNAs，为进一步深入研究microRNA在肺腺癌吉非替尼获得性耐药中的作用及其分子机制提供了实验依据和理论基础。

## Abstract (en)

**Background and objective:**

Acquired gefitinib-resistance was closely related to inefficiency of EGFRTKI treatment in lung adenocarcinoma. However, it was not clear that how microRNAs influenced the acquired gefitinibresistance in lung adenocarcinoma. The aim of this study is to screen and identify the microRNAs correlated with the acquired gefitinib -resistance in lung adenocarcinoma.

**Methods:**

Morphological difference was observed in gefitinib-sensitive lung adenocarcinoma cell line PC9 cell line and gefitinib-resistance lung adenocarcinoma cell line PC9/AB11 cell line derived from PC9 cell line. Cell cycles and doubling time were detected by flow cytometry, IC_50_ of gefitinib was evaluated by MTT assay. The differential microRNAs related to acquired gefitinib-resistance were screened and identified by microRNA array and real-time PCR.

**Results:**

There were obvious morphological differences between PC9 and PC9/AB11 cells. The doubling time, distribution of cell cycle, and the IC_50_ between PC9 and PC9/AB11 were significantly different. In microarray analysis, compared with PC9 cell line, 4 up-regulated microRNAs were found in PC9/AB11 cells, 9 down-regulated microRNAs were found in PC9/ AB11 cells. Real-time PCR revealed thatmiR-138 was significantly down-regulated in PC9/AB11 cells, accord with the microarray.

**Conclusion:**

MicroRNAs are involved in acquired Gefitinib-resistance of lung adenocarcinoma. Our data presented here to provide an experimental basis and theory thereunder for further study of effect and molecule mechanism underlying the acquired gefitinib-resistance of lung cancer.

肺癌是世界上死亡率最高的一种恶性肿瘤，非小细胞肺癌（non-small cell lung cancer, NSCLC）占到了肺癌总数的80%^[1, 2]^。在NSCLC中表皮生长因子受体（epidermal growth factor receptor, EGFR）常存在过度表达和异常激活^[2]^，且EGFR信号通路在癌症发生、发展、转移、凋亡等方面有着重要作用。因此，以阻断EGFR功能为目标的分子靶向治疗逐渐应用到临床^[3]^。吉非替尼（Gefitinib，商品名为易瑞沙，Iressa）可以特异性地抑制EGFR自身磷酸化，阻断其信号传导，从而起到抗肿瘤作用^[4]^，对老年肺腺癌及复发的NSCLC疗效明显^[5, 6]^。近年来，人们发现几乎所有对吉非替尼治疗有效的病例在经过一定时间的缓解期后都会产生吉非替尼的获得性耐药。因此，研究其耐药机制具有重大的理论和临床意义^[7]^。本研究以吉非替尼敏感肺腺癌细胞株PC9与吉非替尼耐药肺腺癌细胞株PC9/AB11为细胞模型，通过microRNA芯片技术分析比较吉非替尼敏感和耐药的肺腺癌细胞株的microRNAs表达谱差异，筛选与肺腺癌吉非替尼获得性耐药相关的microRNAs。

## 1 材料与方法

### 1.1 材料

#### 1.1.1 细胞系

本实验用吉非替尼敏感的人肺癌细胞株PC9细胞，及其衍生的获得性耐药细胞株PC9/AB11细胞均由同济大学附属上海市肺科医院肿瘤研究所惠赠。

#### 1.1.2 试剂与材料

RPIM-1640培养基（Gibco公司），小牛血清（Gibco公司），0.25%胰酶（Gibco公司），吉非替尼片（阿斯利康公司），MTT（Sigma公司），miRNeasy Mini Kit（QIAGEN公司），microRNA芯片（Affymetrix公司）

### 1.2 实验方法

#### 1.2.1 细胞培养

细胞使用含10%小牛血清的RPMI-1640培养基置于37 ℃恒温、5%CO_2_的培养箱中培养，耐药株PC-9/AB11细胞以吉非替尼终浓度为0.05 μmol/L的培养基培养。细胞密度至80%时以0.25%胰酶消化传代。

#### 1.2.2 细胞形态学观察

细胞培养至对数生长期，倒置相差显微镜（Nikon）下观察细胞形态学的差异。

#### 1.2.3 流式细胞术检测细胞周期

取同样处于对数生长期的细胞约1×106个，PBS洗涤，离心，加入PI染色液，FACSAriaTM流式细胞仪（Becton Dickinson公司）检测细胞周期。重复3次。

#### 1.2.4 计数法绘制生长曲线

细胞铺板接种数以7天-10天长满为宜，共设10个点，每24 h计数1个点，每个点设3个重复。利用公式1[Td=T× lg2/lg(N/N0)，Td：倍增时间；T：间隔时间；N：终点细胞数；N0：起点细胞数]计算倍增时间。

#### 1.2.5 MTT法测细胞耐药指数

取状态良好的处于对数生长期的细胞，用0.25%胰酶消化后制成细胞悬液，按每孔8×10^3^个细胞，每孔体积100 μL，接种于96孔培养板中。培养24 h后，依次更换为吉非替尼终浓度为0 μmol/L、0.2 μmol/L、0.5 μmol/L、2 μmol/L、5 μmol/L、10 μmol/L、20 μmol/L、40 μmol/L的培养基，0 μmol/L为对照孔，不加细胞者为调零孔。每浓度设置6复孔。培养72 h后，向每孔加入新鲜MTT（5 mg/mL）20 μL，继续培养4 h后，弃去培养液，每孔加入150 μL DMSO，37 ℃低速震荡10 min，酶联免疫检测仪上测定490 nm波长各孔吸光值（D）。按公式2计算细胞生长抑制率，公式2：细胞生长抑制率=（D_对照组_-D_实验组_）/D_对照组_× 100%。绘制不同浓度下细胞生长抑制率曲线，并用Graphpad Prism 5.0软件计算吉非替尼的半数抑制浓度（IC_50_）。实验重复3次。

#### 1.2.6 microRNA表达谱检测

参照Affymetrix公司推荐方案用Trizol法提取总RNA，将制备好的RNA注入已备好的芯片，用1/2"Tough-Spots盖上两侧隔片，将芯片放入杂交炉，48 ℃，60 rpm孵育16 h，从杂交炉取出芯片，去掉Tough-Spots，提取hybridization cocktail移入新管，降至室温后进行洗脱和染色。利用芯片扫描工作站3000系统（GeneChip 3000 7G，Affymetrix公司）扫描芯片。应用free miRNA QC Tool software初步分析结果。应用MiRNA QC Tool软件进行数据分析，将芯片所有探针组的Signal从小到大排序，去掉最大的2%和最小的2%后，将剩下探针组的平均信号值调整到500。差异miRNA筛选标准：①信号ratio≥2为上调miRNA；②信号ratio≤0.5为下调miRNA。

#### 1.2.7 Real-time PCR检测

随机选取表达具有明显差异的microRNA进行real-time PCR实验。设计待检测microRNA引物，进行逆转录，将逆转录产物进行real-time PCR，分析结果。

### 1.3 统计学分析

应用SPSS 13.0进行统计学分析，计量数据以Mean±SD表示，两样本均值比较采用*t*检验，*P* < 0.05为差异有统计学意义。

## 2 结果

### 2.1 细胞形态学比较

在倒置相差显微镜低倍镜下观察，PC9呈不规则多角形，PC9/AB11呈梭形，细胞接触抑制消失，有少量细胞堆积现象（图 1）。

**1:**
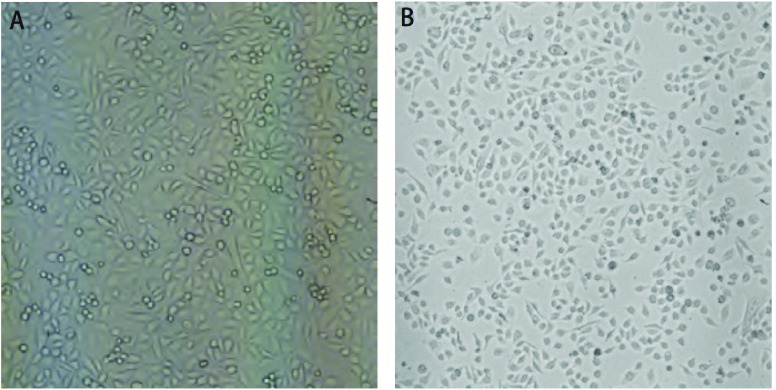
PC9细胞（A）与PC9/AB11细胞（B）形态学比较 The morphology between PC9 cell line (A) and PC9/AB11 cell line (B)

### 2.2 细胞周期测定

PC9与PC9/AB11在G_0_/G_1_期分别为（52.14±0.95）%和（58.36±2.09）%，差异具有统计学意义（*P* < 0.05）；S期分别为（43.24±0.74）%和（36.06± 1.80）%，差异具有统计学意义（*P* < 0.05）；G_2_/M期分别为（4.62±0.74）%和（5.58±0.33）%，两组相比无统计学差异（*P* > 0.05）（图 2）。

**2:**
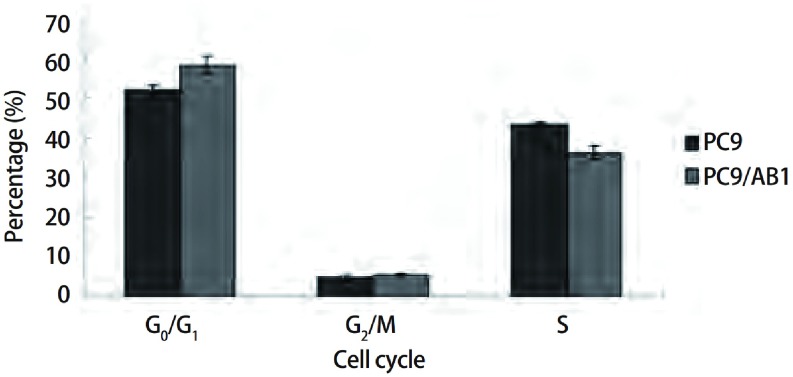
PC9细胞与PC9/AB11细胞的细胞周期分布 Comparison of the distribution of cell cycle between PC9 cell line and PC9/AB11 cell line

### 2.3 绘制生长曲线并计算倍增时间

当细胞处于对数生长期时，计算细胞倍增时间，由公式1计算PC9和PC9/AB11的倍增时间分别是（25.80±1.59）h和（56.05±1.95）h，差异具有统计学意义（*P* < 0.05）（图 3）。

**3:**
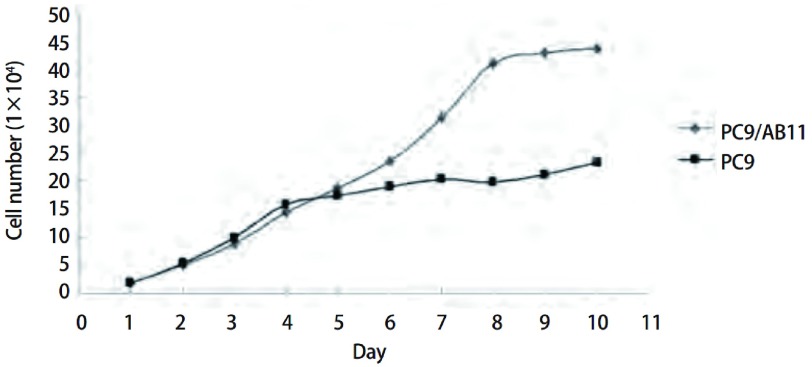
PC9细胞与PC9/AB11细胞的生长曲线 Growth curves between PC9 cell line and PC9/AB11 cell line

### 2.4 吉非替尼对PC9及PC-9/AB11的IC_50_

由公式2计算PC9和PC9/AB11细胞的抑制率，以吉非替尼浓度为因变量，以细胞抑制率为应变量，绘制两种细胞的生长抑制曲线。通过Graphpad Prism 5.0软件计算PC9和PC9/AB11细胞的IC_50_分别为（0.02±0.01）μmol/L和（2.07±0.11）μmol/ L，两者差异具有统计学意义（*P* < 0.05）（图 4）。

**4:**
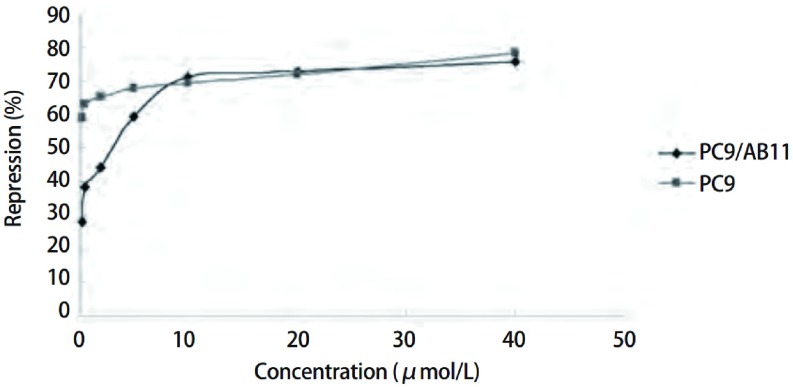
不同浓度吉非替尼对PC9细胞和PC9/AB11细胞的抑制率 The suppressive rates of Gefitinib on PC9 cell line and PC9/AB11 cell line at different concentrations of Gefitinib

### 2.5 MicroRNA芯片结果

将PC9细胞和PC9/AB11细胞分别进行microRNA芯片检测，芯片杂交结果的扫描图如图 5所示。芯片结果经数据处理和统计学分析，PC9细胞与PC9/AB11细胞共有13个microRNA的表达水平具有明显性差异，PC9/AB11细胞相对于PC9细胞高表达的microRNA有4个，低表达的有9个。

**5:**
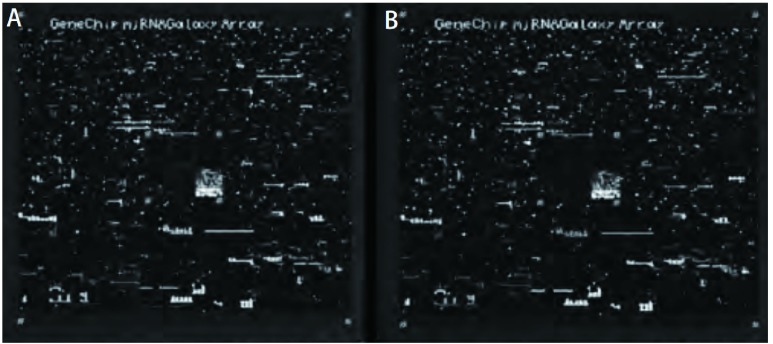
PC9细胞（A）与PC9/AB11细胞（B）microRNA芯片杂交图 PC9 cell line (A) and PC9/AB11 cell line (B) microarray scans

### 2.6 Real-time PCR验证结果

随机抽取芯片结果中的miR-138进行real-time PCR验证，引物设计如表 1所示。real-time-PCR结果显示，miR-138在PC9细胞中的2^-△△Ct^值为（1.005, 0±0.086, 3），PC9/AB11细胞中2^-△△Ct^值为（0.008, 4± 0.000, 6），差异明显（*P* < 0.05）（图 6）。与芯片结果一致。

**1:** 逆转录及real-time PCR中microRNA-138引物序列 The primer sequence of microRNA-138 in reverse transcription and real-time PCR

Primers	Sequence
Stem-ring primer for miR-138	5'-GCGTATCCAGTGCAGGGTCCGAGGTATTCGCACTGGATACGACCGGCCTG-3'
Forward primer for *miR-138* gene	5'-GCAGCTGGTGTTGTGAATC-3'
Forward primer for U6	5'-TGCGGGTGCTCGCTTCGGCAGC-3'
Common reverse primer	5'-CAGTGCAGGGTCCGAGGT-3'

**6:**
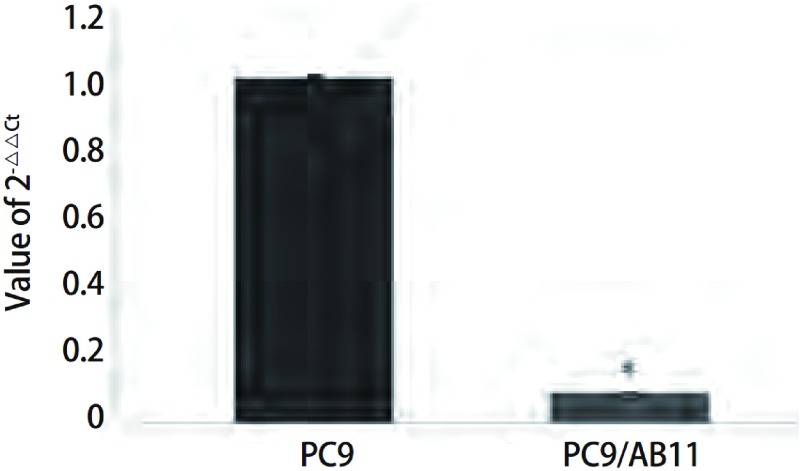
MicroRNA-138在PC9和PC9/AB11中的表达比较 Comparison of the expression level of microRNA-138 between PC9 cell line and PC9/AB11 cell line

## 3 讨论

吉非替尼是针对EGFR酪氨酸激酶抑制剂的靶向治疗药物，对于*EGFR*突变和高拷贝*EGFR*基因的肿瘤具有较好的敏感性^[8, 9]^，患者病情可以得到缓解甚至长期带瘤生存，但是部分患者在经过3个月-7个月的缓解期后出现了病情进展^[10]^，继续应用吉非替尼未能取得任何效果，此类患者出现了获得性耐药。研究^[11]^发现吉非替尼获得性耐药的产生是多种机制作用的结果。Kobayashi等^[12]^首次报道了*EGFR*基因外显子20的C→T的点突变所导致第790位甲硫氨酸取代苏氨酸（T790M）是导致吉非替尼耐药的原因。此后，有多个研究小组^[13, 14]^发现在发生获得性耐药的肿瘤内（具有L858R或delE746-A750突变）检测到了T790M二次突变。这一现象表明，应用吉非替尼治疗可能给予具有T790M突变的肿瘤细胞一种选择性生长优势，从而导致最终出现耐药现象。EGFR信号通路与肿瘤耐药有密切关系，Koizumi等^[15]^在PC-9及PC-9/ZD细胞中分析了EGFR及其衔接蛋白GRB2、SOS和Shc形成的复合物的差别。结果显示，异常的衔接蛋白介导的EGFR到AKT的信号转导可能是PC-9/ZD细胞中吉非替尼获得性耐药的一个机制。Uchida等^[16]^将致癌的*K-Ras*突变体转染入吉非替尼敏感的细胞株内（PC-9和具有EGFR-L858R突变的293T细胞），验证了下游信号通路中的突变导致的信号激活可以导致肺癌中吉非替尼获得性耐药。Jeffrey等^[17]^证实持续活化的PI3K/Akt信号通路在吉非替尼耐药的过程中起到了一定的作用。PTEN的主要作用机制是Akt的去磷酸化作用，可以降低细胞内Akt的水平，发挥与PI-3K相反的作用。She等^[18]^发现PTEN的失活可以导致EGFR信号通路的异常，进而引起吉非替尼耐药。

MicroRNA是一种内源性的非编码的RNA序列，一般由21 nt-23 nt组成单链RNA分子，广泛存在于各种生物体中^[19]^，在转录后水平负性调控基因的表达^[20]^。人们相继发现microRNA参与人体多种生命活动，可以促进细胞增殖^[21]^，对细胞的凋亡通路进行调节^[22]^，参与细胞周期的调控^[23]^，参与人体的代谢过程^[24, 25]^等。MicroRNA不仅参与细胞增殖、分化、凋亡、周期调控等，对肿瘤的化疗耐药也具有重要作用。Meng等^[26]^利用裸鼠构建胆管癌细胞移植瘤模型，在给予吉西他滨处理后有大量的microRNAs表达水平改变，证实microRNA可能参与了肿瘤细胞对化疗药物的反应。有研究^[27]^表明卵巢癌中高表达的miR-214可引起卵巢癌细胞对顺铂耐药，Blower的研究组发现在多种肿瘤细胞中调节miR-21表达水平可导致药物对细胞生长抑制效应的改变，miR-16也可以引起部分药物-细胞对的药效迁移^[28, 29]^，表明MicroRNA是参与肿瘤化疗药耐药的重要因子。microRNA可能也参与了肿瘤的吉非替尼获得性耐药。在乳腺癌细胞SKBr3中转染miR-205的表达载体后，SKBr3细胞对吉非替尼的敏感度增加^[25, 30]^。miR-128b可以靶向EGFR，在多例NSCLC肿瘤标本中可以检测到*miR-128b*基因的杂合性缺失，并且*miR-128b*基因的杂合缺失与吉非替尼用药后的临床效果及生存明显相关^[31]^。在肺癌细胞中过表达miR-let-7a、miR-126和miR-145能够抑制AKT和ERK的活性，并增加肺癌细胞对吉非替尼的敏感度，转染miR-126的表达载体后可使肺癌细胞的耐药指数增加6倍^[32]^。

本实验采用microRNA芯片对吉非替尼敏感株PC9细胞及由其衍生来的耐药细胞株PC9/AB11细胞进行研究，在筛选出来的microRNAs中，多种microRNAs都参与调控细胞增殖、凋亡、周期及肿瘤细胞的侵袭、迁移等过程。MiR-138限制在特定的细胞类型中表达，而他的前体pre-miR-138-2广泛的在所有组织中表达。pre-miR-138-2从细胞核进入细胞质，表明Dicer切割pre-microRNA的过程限制了一定的组织和细胞类型。因此，不同的pre-microRNA可能是控制microRNA功能的机制之一^[33]^。miR-138在神经内管发育过程中，可能靶向于KDM6B的表达^[34]^；microR NA-138直接靶作用于EID-1的3'UTR区，负性调控机体的脂肪细胞生成过程^[35]^；还可以促进少突细胞的分化和髓鞘形成^[36]^；在先天无脑畸形的患儿组织里，miR-138明显下调，可能与先天无脑畸形的形成有关^[37]^。MicroRNA可以在转录后的水平进行调节。Liu等^[38]^研究表明，miR-138在头颈部肿瘤中可以抑制肿瘤侵袭促进凋亡；Mitomo^[39]^研究表明，miR-138可能通过作用于端粒酶逆转录酶参与了甲状腺癌的发生。Zhao等^[40]^的研究证实miR-138也参与了肿瘤化疗耐药，miR-138可以显著下调多重耐药基因的表达，并逆转白血病细胞株的多重耐药。

本研究中，我们的实验结果提示microRNA对肺腺癌细胞的吉非替尼获得性耐药的产生可能具有重要作用。在此研究基础上我们将对microRNA对肺癌细胞的吉非替尼获得性耐药产生机制进行深入研究。本研究为全面、深入研究肺腺癌靶向治疗耐药的分子机制，筛选可预测肺腺癌靶向治疗疗效的microRNAs标志物提供了实验基础。

## References

[1] Jemal A, Tiwari RC, Murray T (2004). Cancer statistics. CA Cancer J Clin.

[2] Rusch V, Baselga J, Cordon-Cardo C (1993). Differential expression of the epidermal growth factor receptor and its ligands in primary non-small cell lung cancers and adjacent benign lung. Cancer Res.

[3] Ciardiello F, Tortora G (2001). A novel approach in the treatment of cancer: targeting the epidermal growth factor receptor. Clin Cancer Res.

[4] Paez JG, Janne PA, Lee JC (2004). EGFR mutations in lung cancer: correlation with clinical response to gefitinib therapy. Science.

[5] Liu LX, Li L, Zhou QH (2004). Iressa for the non-small cell lung cancer patients who failed prior chemotherapy and radiotherapy. Chin J Lung Cancer.

[6] Wang Y, Chu DT, Sun Y (2008). Clinic outcome of gefitinib in sixty-nine elderly patients with lung adenocarcinoma. Chin J Lung Cancer.

[7] Morgillo F, Kim WY, Kim ES (2007). Implication of the insulin-like growth factor-IR pathway in the resistance of non-small cell lung cancer cells to treatment with gefitinib. Clin Cancer Res.

[8] Riely GJ, Politi KA, Miller VA (2006). Update on epidermal growth factor receptor mutations in non-small cell lung cancer. Clin Cancer Res.

[9] Tracy S, Mukohara T, Hansen M (2004). Gefitinib induces apoptosis in the EGFRL858R non-small-cell lung cancer cell line H3255. Cancer Res.

[10] Hoshi S, Yamaguchi T, Kono C (2004). Recurrence of non-small cell lung cancer after successful treatment with gefitinib-report of three cases. Gan To Kagaku Ryoho.

[11] Xu Y, Chen J, Zhou Q (2010). Acquired resistance of lung adenocarcinoma to EGFR-tyrosine kinase inhibitors gefitinib and erlotinib. Cancer Biol Ther.

[12] Kobayashi S, Boggon TJ, Dayaram T (2005). *EGFR* mutation and resistance of non-small cell lung cancer to gefitinib. N Engl J Med.

[13] Gow CH, Shih JY, Yu CJ (2005). Acquired gefitinib-resistant mutation of EGFR in a chemonaive lung adenocarcinoma harboring gefitinib-sensitive mutation L858R. PLoS Med.

[14] Uramoto H, Sugio K, Yasumoto K (2006). Resistance to gefitinib. Int J Clin Oncol.

[15] Koizumi F, Shimoyama T, Nishio K (2005). Establishment of a human nonsmall cell lung cancer cell line resistant to gefitinib. Int J Cancer.

[16] Uchida A, Hirano S, Kitao H (2007). Activation of downstream epidermal growth factor receptor (EGFR) signaling provides gefitinib-resistance in cells carrying EGFR mutation. Cancer Sci.

[17] Wakeling AE, Guy SP, Woodburn JR (2002). ZD1839 (Iressa): an orally active inhibitor of epidermal growth factor signaling with potential for cancer therapy. Cancer Res.

[18] She QB, Solit D, Moasser MM (2003). Resistance to gefitinib in PTEN-null HER-overexpressing tumor cells can be overcome through restoration of PTEN function or pharmacologic modulation of constitutive phosphatidylinositol 3'-kinase/Akt pathway signaling. Clin Cancer Res.

[19] Grishok A, Pasquinelli AE, Conte D (2001). Genes and mechanisms related to RNA interference regulate expression of the small temporal RNAs that control C. elegans developmental timing. Cell.

[20] Khvorova A, Reynolds A, Jayasena SD (2003). Functional siRNAs and miRNAs exhibit strand bias. Cell.

[21] Hipfner DR, Weigmann K, Cohen SM (2002). The bantam gene regulates Drosophila growth. Genetics.

[22] Cheng AM, Byrom MW, Ford LP (2005). Antisense inhibition of human miRNAs and indications for an involvement of miRNA in cell growth and apoptosis. Nucleic Acids Res.

[23] Linsley PS, Schelter J, Burchard J (2007). Transcripts targeted by the microRNA-16 family cooperatively regulate cell cycle progression. Mol Cell Biol.

[24] Poy MN, Eliasson L, Krutzfeldt J (2004). A pancreatic islet-specific microRNA regulates insulin secretion. Nature.

[25] Krutzfeldt J, Rajewsky N, Braich R (2005). Silencing of microRNAs *in vivo* with 'antagomirs'. Nature.

[26] Xi Y, Formentini A, Chien M (2006). Prognostic values of microRNAs in colorectal cancer. Biomark Insights.

[27] Yang H, Kong W, He L (2008). MicroRNA expression profiling in human ovarian cancer: miR-214 induces cell survival and cisplatin resistance by targeting PTEN. Cancer Res.

[28] Blower PE, Verducci JS, Lin S (2007). MicroRNA expression profiles for the NCI-60 cancer cell panel. Mol Cancer Ther.

[29] Blower PE, Chung JH, Verducci JS (2008). MicroRNAs modulate the chemosensitivity of tumor cells. Mol Cancer Ther.

[30] Pao W, Wang TY, Riely GJ (2005). *KRAS* mutations and primary resistance of lung adenocarcinomas to gefitinib or erlotinib. PLoS Med.

[31] Weiss GJ, Bemis LT, Nakajima E (2008). EGFR regulation by microRNA in lung cancer: correlation with clinical response and survival to gefitinib and EGFR expression in cell lines. Ann Oncol.

[32] Zhong M, Ma X, Chen L (2010). MicroRNAs reduce tumor growth and contribute to enhance cytotoxicity induced by gefitinib in non-small cell lung cancer. Chem Biol Interact.

[33] Obernosterer G, Leuschner PJ, Martinez J (2006). Post-transcriptional regulation of microRNA expression. RNA.

[34] Ichi S, Costa FF, Bischof JM (2010). Folic acid remodels chromatin on Hes1 and Neurog2 promoters during caudal neural tube development. J Biol Chem.

[35] Yang Z, Bian C, Zhou H (2011). MicroRNA hsa-miR-138 inhibits adipogenic differentiation of human adipose tissue-derived mesenchymal stem cells through adenovirus EID-1. Stem Cells Dev.

[36] Dugas JC, Cuellar TL, Scholze A (2010). Dicer1 and miR-219 Are required for normal oligodendrocyte differentiation and myelination. Neuron.

[37] Zhang Z, Chang H, Li Y (2010). MicroRNAs: potential regulators involved in human anencephaly. Int J Biochem Cell Biol.

[38] Liu X, Jiang L, Zhou X (2009). MicroRNA-138 suppresses invasion and promotes apoptosis in head and neck squamous cell carcinoma cell lines. Cancer Lett.

[39] Mitomo S, Maesawa C, Ogasawara S (2008). Downregulation of miR-138 is associated with overexpression of human telomerase reverse transcriptase protein in human anaplastic thyroid carcinoma cell lines. Cancer Sci.

[40] Zhao X, Yang L, Ruan J (2010). miR-138 might reverse multidrug resistance of leukemia cells. Leuk Res.

